# Barriers to Postdischarge Smartphone App Use Among Patients With Traumatic Rib Fractures

**DOI:** 10.2196/52726

**Published:** 2024-05-31

**Authors:** Margaret T Berrigan, Brendin R Beaulieu-Jones, Rachel Baines, Seth Berkowitz, Heather Evans, Gabriel A Brat

**Affiliations:** 1 Department of Surgery Beth Israel Deaconess Medical Center Boston, MA United States; 2 Department of Biomedical Informatics Harvard Medical School Boston, MA United States; 3 Department of Radiology Beth Israel Deaconess Medical Center Boston, MA United States; 4 Department of Surgery Medical University of South Carolina Charleston, SC United States

**Keywords:** mobile health, smartphone app, electronic health record, postdischarge monitoring, implementation science, mHealth, app, apps, application, applications, digital health, smartphone, smartphones, mobile phone

## Abstract

Rib fractures commonly result from traumatic injury and often require hospitalization for pain control and supportive pulmonary care. Although the use of mobile health technology to share patient-generated health data has increased, it remains limited in patients with traumatic injuries. We sought to assess the feasibility of mobile health tracking in patients with rib fractures by using a smartphone app to monitor postdischarge recovery. We encountered patient, institutional, and process-related obstacles that limited app use. The success of future work requires the acknowledgment of these limitations and the use of an implementation science framework to effectively integrate technological tools for personalized trauma care.

## Introduction

Rib fractures are common among adults and frequently result from traumatic injury. Management often requires hospitalization and focuses on pain control and supportive pulmonary care [1]. Patient-generated health data sharing and mobile health technology use have increased with expanded technical ability and smartphone uptake. Most existing examples target patients enrolled in an elective outpatient setting, but some include acute inpatient enrollment [2-6]. Understanding that electronic data exchange has historically been limited among patients with traumatic injuries, we sought to assess the feasibility of mobile health tracking in patients with rib fractures by using a smartphone app to monitor postdischarge recovery.

## Methods

### Overview

**Figure 1:**
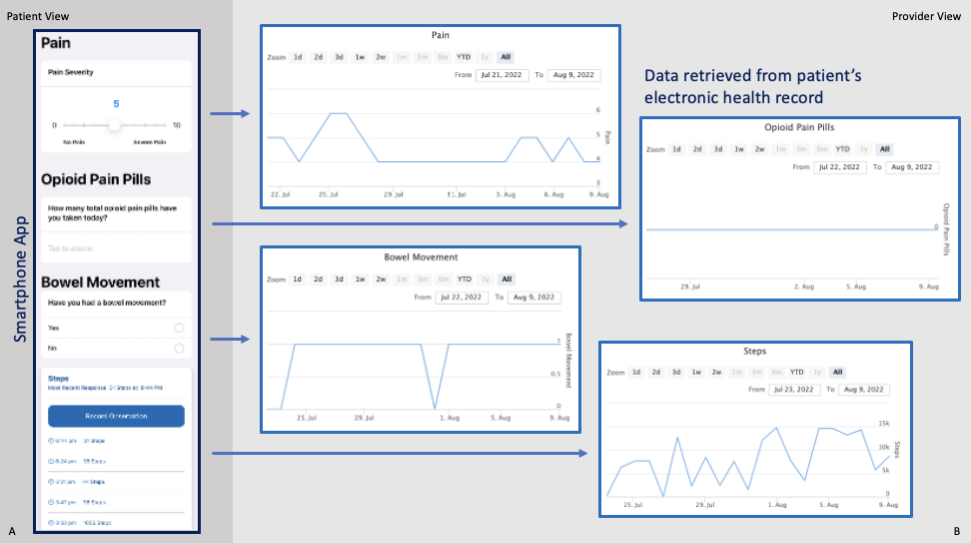
Smartphone app and electronic health record data retrieval: (A) the patient-facing smartphone app, including data entry for pain severity, use of opioid pain medication, frequency of bowel movements, and step count, and (B) the corresponding data review interface accessible to the provider within the electronic health record. YTD: year to date.

All consecutive adult patients with ≥1 rib fracture admitted to the trauma service from July to August 2022 were approached. Interested and eligible patients were enrolled and had the app (pilot available only in English and for iOS devices) loaded on their phones. The app was developed by the authors for use at a single, large, urban, academic, level I trauma center. After discharge, patients actively entered data on pain severity, opioid medication use, and bowel movement frequency. Patient step count was passively collected by the app. Data were stored and accessible in the electronic health record (EHR; Figure 1).

### Ethical Considerations

This quality improvement effort was undertaken with the support and within the standard clinical practice of the trauma surgery service. All patients admitted with rib fractures were offered the use of the hospital-approved postdischarge smartphone app; patients could stop use or remove the app from their phones at any time. Patients were not compensated for app use. Patients were informed that their providers would be able to access data they entered into the app solely via their existing EHR and that the app was not a means for urgent communication. A review of the quality improvement project was performed; no separate institutional review board approval or consent was required, and only the clinical team had access to identifiable data.

## Results

During the pilot period, 25 patients with rib fractures were admitted to the trauma service (Table 1).

Of the 25 patients, 2 (8%) participated in the study. The other 23 (92%) patients did not participate for the following reasons: 6 (24%) had prohibitively low functional status (delirium, dementia, or traumatic brain injury), 8 (32%) did not have an iOS device (used an Android device or feature phone or did not have a mobile phone), 2 (8%) did not speak English, 5 (20%) declined participation, and 2 (8%) could not download the app while admitted due to patient or team member unavailability and the need for in-hospital enrollment. Both patients who used the app had multiple injuries and recovered to their preinjury functional status without complication. They reported data for 2 days and 2 weeks after discharge, respectively, and disclosed no problems with app use.

**Table 1:** Patient characteristics and results of attempted app download. The age, sex, mechanism of injury, and injury burden including the presence of rib fractures for patients approached for app download are shown. The results of attempted app download are described, including common barriers to app use.

Variable					Value
**Characteristics of patients admitted with rib fractures in a 1 month period (n=25)**					
	Age (years), median (IQR)			69 (25-91)	
	**Sex, n (%)**				
		Male	17 (68)		
		Female	8 (32)		
	**Mechanisms of injury, n (%)**				
		Falls	13 (52)		
		Vehicular accidents	7 (28)		
	**Injuries, n (%)**				
		Rib fractures with or without associated pneumothorax	7 (28)		
		Rib fractures and other traumatic injury	18 (72)		
**Results of attempted app download, n (%)**					
	Downloaded and used the app			2 (8)	
	Prohibitively low functional status			6 (24)	
	No iPhone			8 (32)	
	Non-English speaker			2 (8)	
	Declined participation			5 (20)	
	Not available at attempt to download			2 (8)	

## Discussion

We developed a novel smartphone app to collect patient-reported postdischarge data within the existing EHR. However, we observed patient, institutional, and process-related obstacles that limited study participation by patients with traumatic rib fractures. These limitations appear to extend to the general trauma population, as we anecdotally encountered similar results when we engaged patients admitted to the trauma service with injuries other than rib fractures.

Patients with traumatic injuries, often from vulnerable populations, present unexpectedly and urgently. It is not possible to meet them in clinic prior to presentation for planned enrollment. Further, patients with traumatic injuries may not have access to a smartphone or have the ability to use a postdischarge smartphone app. Faced with the stress of injury, patients may decline to participate and require additional incentives to use such technology. This reluctance may be amplified when it is unclear how their participation will aid recovery.

We did not observe technical or user-reported issues with app use. However, the onboarding process was burdensome to team members and patients. Streamlining this within the existing clinical and technological infrastructure might improve onboarding but requires substantial institutional investment in EHR customization, as well as the allocation of already limited human resources for enrollment. To reach more patients, an Android app is being developed.

Smartphone apps for the collection of patient-generated data represent a new opportunity for postdischarge management of patients who sustain traumatic injury. The success of future work requires the acknowledgment of the described limitations. Researchers should use an implementation science framework [7] to better understand the requirements and motivations of key stakeholders, leverage technology to understand the postdischarge patient experience, and integrate such tools effectively for personalized trauma care.

## Abbreviations

EHR: electronic health record

## Data Availability

All shareable data are included in this paper; additional data are not publicly available given the potential for patient reidentification.
